# A Review on Endoscopic Management of Acute Cholecystitis: Endoscopic Ultrasound-Guided Gallbladder Drainage and Endoscopic Transpapillary Gallbladder Drainage

**DOI:** 10.3390/medicina60020212

**Published:** 2024-01-26

**Authors:** Albert P. Manudhane, Matthew D. Leupold, Hamza W. Shah, Raj Shah, Samuel Y. Han, Peter J. Lee, Jordan J. Burlen, Georgios I. Papachristou, Somashekar G. Krishna

**Affiliations:** 1 Division of Gastroenterology, Hepatology and Nutrition, The Ohio State University Wexner Medical Center, 395 W. 12th Avenue, Suite 262, Columbus, OH 43210, USA; albert.manudhane@osumc.edu (A.P.M.);; 2 Department of Internal Medicine, The Ohio State University Wexner Medical Center, Columbus, OH 43210, USA

**Keywords:** acute cholecystitis, endoscopic transpapillary gallbladder drainage, endoscopic ultrasound-guided gallbladder drainage, percutaneous cholecystostomy, laparoscopic cholecystectomy, lumen-apposing metal stent, AXIOS, double-pigtail plastic stent

## Abstract

A percutaneous cholecystostomy tube (PCT) is the conventionally favored nonoperative intervention for treating acute cholecystitis. However, PCT is beset by high adverse event rates, need for scheduled reintervention, and inadvertent dislodgement, as well as patient dissatisfaction with a percutaneous drain. Recent advances in endoscopic therapy involve the implementation of endoscopic transpapillary drainage (ETP-GBD) and endoscopic ultrasound-guided gallbladder drainage (EUS-GBD), which are increasingly preferred over PCT due to their favorable technical and clinical success combined with lower complication rates. In this article, we provide a comprehensive review of the literature on EUS-GBD and ETP-GBD, delineating instances when clinicians should opt for endoscopic management and highlighting potential risks associated with each approach.

## 1. Introduction

Acute cholecystitis—primarily caused by gallstone obstruction of the cystic duct—involves the inflammation of the gallbladder. Only a small fraction of cases, approximately 5–10%, occur in the absence of gallstones [1,2]. Factors that are correlated with calculous gallbladder pathology include female gender, obesity, pregnancy, and a sedentary lifestyle that contains food with excessive fat and low fiber. Susceptibilities for acalculous cholecystitis include acute critical illness, male sex, advanced age over 50, presence of HIV, and use of total parenteral nutrition. Classic symptoms of acute cholecystitis include nausea, vomiting, and right upper quadrant abdominal pain [1,2].

The diagnosis is typically made with ultrasound or computerized tomography (CT) which demonstrates gallbladder wall fluid, thickening, or distension. Fat stranding around the gallbladder wall is sometimes seen on CT [1,3]. If ultrasound and/or CT imaging is equivocal for acute cholecystitis, hepatobiliary scintigraphy (HIDA) may be utilized to corroborate the diagnosis. Laboratory tests may reveal elevations in the white blood cell count, alkaline phosphatase, and total bilirubin. Affecting over 200,000 individuals in the US per year, acute cholecystitis is typically treated with antibiotics and eventual surgical cholecystectomy. Performing laparoscopic cholecystectomy within 1–3 days of presentation is correlated with a reduction in postoperative adverse events, a shorter hospital stay, and decreased hospital expenses compared to surgeries conducted later during the same admission [1,2]. 

If acute cholecystitis is left untreated without surgery or decompression, potential long-term sequelae may include complications such as perforation, gangrenous cholecystitis, and the formation of a fistula between the gallbladder and bowel [4]. In critically ill patients facing an unacceptably high perioperative risk, clinicians must explore all non-surgical management options. Traditional non-surgical approaches typically involve the use of antibiotics alone or in conjunction with percutaneous cholecystostomy (PCT) [1,5]. If performed, PCT provides decompression until the patient becomes more stable for surgery and is not typically a definitive treatment [4]. More recent therapy for nonsurgical candidates includes endoscopic ultrasound-guided gallbladder drainage (EUS-GBD) involving the insertion of a lumen-apposing metal stent (LAMS) between the gallbladder and stomach (cholecystogastrostomy) or between the gallbladder and duodenum (cholecystoduodenostomy). In addition, another alternative to PCT involves endoscopic transpapillary gallbladder drainage (ETP-GBD) which is accomplished by the placement of a double pigtail plastic stent (DPPS) into the gallbladder that extends through the cystic duct and common bile duct (CBD) into the small bowel lumen [1,6,7,8]. The American Gastroenterology Association (AGA) recommends consideration of endoscopic management for acute cholecystitis prior to PCT in a carefully selected patient population [9]. 

EUS-GBD and ETP-GBD can be performed through the use of moderate sedation, monitored anesthesia care (typically with propofol), or general anesthesia. If the endoscopist is comfortable with providing moderate sedation, medications such as fentanyl, midazolam, and dexmedetomidine can be chosen depending on patient hemodynamics and risk of respiratory depression [10,11,12]. Often times, anesthesiology is tasked with providing sedation due to their expertise in managing more unstable patients. Proceduralists frequently prefer propofol or general anesthesia for decreased patient movement, optimization of windows, and subsequent ease of access to structures important for successful procedure completion [6,11].

This review will outline the standard approach to managing acute cholecystitis, provide a thorough overview of non-surgical, non-endoscopic management techniques like PCT, discuss the indications for endoscopic therapy, delve into the various techniques used for endoscopic interventions, compare and contrast EUS-GBD and ETP-GBD, and finally, address the long-term management of patients treated endoscopically.

## 2. Standard Management of Acute Cholecystitis 

### 2.1. Management: Surgery and Antimicrobial Therapy

Upon diagnosis of acute cholecystitis, antibiotics are initiated. In cases of uncomplicated acute cholecystitis, laparoscopic cholecystectomy is usually performed shortly after clinical improvement and often within 1–3 days of hospital admission. This approach decreases the risk of complications and shortens hospital stays. If the patient presents with complicated acute cholecystitis, surgery may be postponed for a week or longer, depending on the severity of the systemic illness. One study found a 11.8% risk of adverse events in patients who had cholecystectomy within one day of admission, compared to a 34.4% complication rate in those receiving surgery 7–45 days following presentation [1,5]. Another large retrospective study from the US found that 25% of geriatric patients with acute cholecystitis did not ultimately receive surgery during their index hospitalization [13].

### 2.2. Percutaneous Cholecystostomy (PCT) and Emerging Endoscopic Therapy

Traditionally, PCT has been employed to decompress the gallbladder in nonoperative management of acute cholecystitis. Originally developed by interventional radiologists in the 1980s, PCT offered a non-surgical solution for patients at high risk for surgery [1,14]. Nevertheless, PCT has notable drawbacks. Biliary leaks, hemobilia, pneumothorax, and bowel perforation are among the well-known complications of PCT. Furthermore, skin irritation, persistent pain at the tube site, and inconvenience of having an external drain are common complains. Tube dislodgement is a frequent issue which often necessitates reintervention. These drawbacks underscore the importance of careful consideration of the risks and benefits of this procedure before proceeding with it [9].

The morbidity associated with PCT is notably high, with estimates ranging from 50–75% according to one study [9]. PCT diverts bile from its normal digestive path in the gastrointestinal tract, where it is normally reabsorbed in the ileum and colon. As a result, the bile acid pool size is altered, and the liver is burdened with producing more bile to compensate [15,16]. Additionally, PCT is not possible when there are loops of bowel in the anticipated path of the procedure, or when the patient has significant ascites [14]. After PCT placement, the external catheter must remain in place until the tract matures, usually taking around four weeks, but potentially longer—often times months if not years—which can be a burden for the patient [14,17]. Compared to EUS-GBD, PCT is associated with a higher incidence of adverse events and readmission rates, largely due to catheter malfunction [17]. Endoscopic management offers several benefits over PCT, including mitigating some of the risks and limitations of PCT, and is gaining traction as the preferred approach. Despite the risks and limitations of PCT, it is still recommended over EUS-GBD or ETP-GBD when imaging suggests gallbladder perforation, or when a patient is deemed too high risk for endoscopic treatment [9].

## 3. Endoscopic Transpapillary Gallbladder Drainage (ETP-GBD)

### 3.1. Brief Overview of Performing the Procedure

In the early 1990s, authors began publishing reports of cystic duct stenting for the management of acute cholecystitis. Early methods were unrefined and less successful, with clinical improvement noted in only 65% of patients [6,18]. Generally speaking, completion of ETP-GBD involves ERCP with biliary cannulation and biliary sphincterotomy, removal of any debris from the common bile duct, and cannulation of the cystic duct and guidewire progression into the gallbladder. Next, small caliber transpapillary 5–10 French double pigtail stents are introduced into the gallbladder lumen extending through the cystic duct and common bile duct with the distal end placed in the duodenum. At some centers, an initial ERCP is performed with one stent therapy, and the patient is recalled in 4–8 weeks for a repeat ERCP to perform stent removal and replacement with two side-by-side stents. After the placement of one or two double pigtail stents, the devices often remain in place indefinitely unless complications arise necessitating removal [6,19,20]. 

### 3.2. ETP-GBD: Technical Success, Clinical Success, and Additional Review of Studies

Cannulation of the cystic duct can prove challenging, which may limit the technical success of ETP-GBD. Proximal cystic duct direction, the existence of cystic or common bile duct stones, and severe gallbladder wall inflammation may make this procedure more difficult to perform. In a single center in Japan, a review of 323 cases showed that technical success was achieved in 235 instances, accounting for a success rate of 72.8% [21]. On a broader literature review, technical success in experienced centers ranges from 63.6% to 100% [6,22]. In another center with four years or less of experience in ETP-GBD, the procedure was initially successfully completed only 50% of the time. However, their performance improved to 89% during the subsequent five-year period [23]. Hence, endoscopist and center experience correlate with technical success. When attempts to cannulate the cystic duct using the first-line guidewire method are unsuccessful, employing a direct cholangioscopy system can increase the likelihood of success [24].

In a study led by Sato et al., which involved 242 patients, the median age of the participants was 74 years, and gallstones were identified as the causative factor in 83% of cholecystitis cases. Clinical success was defined by the resolution of fever, relief from abdominal pain, improvements in infection-related laboratory parameters, and a notable reduction in liver enzyme levels. They found a clinical success rate of 93% [25]. In a recent retrospective study covering 10 years of data at a single center, technical and clinical success rates were 84.6% (198/234) and 97.4% (193/198), respectively. Over a median follow-up period of 564 days, Kaplan–Meier analysis revealed biliary event-free rates of 99% at 6 months, 92% at 1 year, and 76% at ≥2 years. These studies highlight the effectiveness of ETP-GBD as a biliary drainage method for selected patients, especially those unable to undergo cholecystectomy or with deferred cholecystectomy plans [26]. 

A recent meta-analysis evaluating ETP-GBD included 21 studies and 1307 patients (61.4% male, 38.6% female, median age 68.4 years). Technical success was achieved in 82.6% of cases, clinical success obtained in 94.9% of individuals who successfully completed the procedure, and complications occurred in 8.8% of participants. The most common complications noted in this meta-analysis included postprocedural bleeding (1.0%), perforation (0.8%), peritonitis/bile leak (0.5%), pancreatitis (2.0%), stent obstruction (0.4%) and abnormal stent migration (1.3%). Six studies in this analysis measured the rate of recurrent cholecystitis, with a pooled event rate of 1.5% [27]. 

In a single-center study by Kedia et al., patients who underwent endoscopic gallbladder drainage experienced a significantly shorter hospital stay (8.7 days) and a faster improvement of symptoms (1.6 days) compared to those who received PCT. Furthermore, the adverse event rate was significantly lower in the endoscopic drainage group (13.3%) than in the PCT cohort (39.5%). Interestingly, ETP-GBD was used as the primary endoscopic approach, with EUS-GBD only being performed when ETP-GBD was not feasible. This study demonstrated that when endoscopic drainage is pursued, there are additional means of drainage if one maneuver fails [16].

### 3.3. ETP-GBD: Complications

Complications of ETP-GBD include stent migration or occlusion, pancreatitis, post-sphincterotomy bleeding, gallbladder injury or perforation, cystic duct injury with potential bile leak, postprocedural pain, pericholecystic fluid collection, and recurrent cholecystitis [6,26,27]. One study found that the cystic duct injury complication had a significant correlation with failure to complete the procedure successfully (Odds ratio [OR] of 11, 95% confidence interval [CI]—3.9–29) [25]. With an incidence rate of 9.2%, the severity of cystic duct injury may vary, with complete transection posing the greatest risk for bile leak and peritonitis [25,28]. Prophylactic stenting over the perforation site can decrease the risk of bile leak, as the stent acts as a barrier to prevent bile from escaping. Lavage and aspiration of gallbladder fluid can reduce the bile volume in the gallbladder, which further decreases the risk of bile leak. While cystic duct injury does not preclude successful ETP-GBD, caution should be exercised, and strict monitoring for bile leak is essential [28].

Placing the stent in the distalmost portion of the gallbladder reduces the risk of stent migration due to decreased inflammation-related tension [6]. As younger patients, females, those with sphincter of Oddi dysfunction, prior post-ERCP pancreatitis, or difficult biliary cannulation history are at higher risk of ERCP-associated adverse events, including post-ERCP pancreatitis, endoscopists may avoid ETP-GBD in such patients to minimize the potential for complications.

Prophylactic pancreatic duct stenting (especially in the setting of inadvertent pancreatic duct cannulation), rectal indomethacin, and lactated ringer’s administration may reduce the risk of post-ERCP pancreatitis, and subsequently, the overall ETP-GBD complication rate [29,30].

## 4. Endoscopic Ultrasound-Guided Gallbladder Drainage (EUS-GBD)

### 4.1. EUS-GBD: Introduction

An early approach for EUS-GBD was initially published by Baron and Topazian in 2007. Since its early days involving the use of pigtail biliary stents, EUS-GBD has evolved significantly over the last decade. Refs. [31,32,33] Improved outcomes and reduced risks (due to refined techniques and stent improvement) have led to the growing popularity of EUS-GBD. The use of double-pigtail stents for EUS-GBD fell out of favor as self-expandable metal stents (SEMS) were introduced, due to their lower risk of biliary leak. However, more recently, LAMS (lumen-apposing metal stents) are considered superior to SEMS because of their reduced risk of stent migration, attributed to their flanges and shorter length. Additionally, the integrated cystotome with the LAMS enables EUS-GBD to be performed in a single-stage procedure [7,9]. The AGA recommended prioritizing LAMS over other stent options in its most recent guidelines [9]. Although using LAMS for EUS-GBD was previously considered off-label, in 2023 the AXIOS LAMS (Boston Scientific Corporation) stent was recently reclassified by the FDA through the 513(f)(2) pathway which allows it to be promoted for gallbladder drainage [9,34,35].

### 4.2. EUS-GBD: Brief Overview of Performing the Procedure

To perform the procedure, the endoscopist must first use EUS to determine if there are no vessels or other structures between the gallbladder and gastric antrum or duodenal bulb. The gallbladder should ideally be within 10–20 mm from the lumen where the EUS probe is located. The gallbladder neck is more fixed in position and closer to the duodenal bulb and is an ideal target for stent placement with a transduodenal approach if possible. The gallbladder body is typically near the gastric antrum and is often the best target for transgastric LAMS placement [7,36,37,38].

Catheter access is obtained within the gallbladder, typically with cautery enhanced LAMS. The distal flange is then deployed within the gallbladder and approximated to the gallbladder wall. The proximal flange is then deployed within the bowel or stomach lumen. The ESGE recommends placing the LAMS in the duodenum (as opposed to gastric lumen) to decrease the risk of stent occlusion and malfunction. Alternatively, if the LAMS does not have an integrated cautery device, the procedure is accomplished by first introducing a 19G needle into the gallbladder, introducing a guidewire into the gallbladder lumen, and performing serial dilation until eventually the LAMS is placed. Subsequently, balloon dilation of the stent can be performed to expand the tract for passage of an upper endoscope, which allows for visualization of the gallbladder if desired. Using a cautery-enhanced LAMS appears to be less risky due to fewer procedural steps [4,7,36,37,38]. After stent placement, a trial of liquid diet is performed for 1–2 days, which may later be liberalized to a regular diet. While many endoscopists recommend a low residue diet post-procedure to minimize risk of stent malfunction, there is a dearth of data supporting this [4].

### 4.3. EUS-GBD: Technical Success, Clinical Success, and Long-Term Management

For experienced endoscopists, technical and clinical success for EUS-GBD is quite favorable. Oh et al. performed a retrospective review of 76 patients at their institution and found a technical and clinical success of 99% and 99%, respectively, when the procedure was performed by 3 skilled endoscopists. Their study reviewed charts between 2010 and 2014 and used SEMS to complete EUS-GBD [39]. Higa et al. performed a retrospective chart review of 40 EUS-GBD cases at their institution; their procedures were performed using LAMS between 2013 and 2018 by four expert endoscopists. They found a technical success rate of 97.5% and a clinical success rate of 95% [40]. Reported technical issues with EUS-GBD include mechanical problems with LAMS deployment, issues related to small gallbladder size and subsequent inability to perform deep insertion of the guidewire or stent, and inability to find a safe window for the procedure. Issues hindering clinical success could include abnormal stent migration or occlusion [41,42].

Typically, the LAMS tract is mature in 28–35 days, after which the stent may be removed if necessary. Some endoscopists routinely keep the stent in position for 90 days prior to consideration of removal in an effort to decrease the likelihood of complications such as acute cholecystitis or bile leak [42]. In other instances, gastroenterologists may elect to keep the stent in place permanently. Another approach involves exchanging the LAMS with a pigtail stent after the tract has developed. Two studies found no stent-related issues at one-year follow-up in patients who did not have LAMS removal. There is no expert consensus regarding when the LAMS needs to be removed after EUS-GBD. Potential long-term adverse events from stent retention include late bleeding and breakdown of stent coating material leading to overgrowth of tissue and subsequent obstruction or difficulty with stent removal [4,40,42].

Martinez-Moreno et al. recently published a study in which 50 patients undergoing EUS-GBD via LAMS were not scheduled for routine stent removal and were followed for a median period of 25 months. Over this time period, a total of 13 (26%) experienced complications, most of which were mild. Biliary complications were the most common (39%), and 78% of these cases were improved with repeat endoscopic treatment. Stent-related complications were experienced in 11 patients (22%), at a median time period of 674 days post-procedure. These included acute cholecystitis, abnormal LAMS migration, gastric outlet obstruction, and abnormal stent ingrowth. Yet, the study authors concluded that it may be safe to leave LAMS in place without planned removal [43].

### 4.4. EUS-GBD: Additional Review of Literature

Typically, when EUS-GBD is pursued for nonsurgical management of acute cholecystitis, it is the initial procedure performed for gallbladder drainage and decompression. However, some institutions have a shortage of experienced endoscopists who can perform the procedure urgently. One retrospective study measured the outcomes of 15 patients who first underwent transhepatic PCT for acute cholecystitis and subsequently received EUS-GBD with SEMS during a median duration of 2 weeks following PCT. The authors found technical and clinical success to be 93.3%, with an adverse event rate of 20% (1 episode of pneumoperitoneum, 1 episode of stent migration, and 1 recurrence of cholecystitis). This study demonstrates how EUS-GBD may be performed in a stepwise fashion after PCT. Such an approach can be most useful for smaller institutions where PCT could be performed urgently and patents could be referred to tertiary centers for endoscopic drainage [44]. 

In a recent (2023) prospective multicenter study of EUS-GBD with LAMS, 30 patients underwent EUS-guided LAMS placement. In this study, 67% of patients had transduodenal drainage performed, 27% had LAMS placed transgastrically, and in 7% of cases the patient did not receive a stent due to technical limitations. On average, acute cholecystitis improvement occurred within 1–2 days in patients with LAMS, and all of them experienced symptom relief. In this study, adverse events included diarrhea, fatigue, sepsis, pleural effusion, abdominal pain, electrolyte dysfunction, and stent-related bleeding. In cases where repeat endoscopy was performed for stent removal, all patients were found to have a patent LAMS. Notably, only 68% of patients who received LAMS underwent stent removal. Stent retention was due to various factors, including patient preference to avoid a repeat procedure, a high risk associated with repeat procedures due to age and comorbidities, and patient mortality [45].

When compared to PCT, EUS-GBD has been found to have enhanced clinical success and decreased rates of reintervention, with comparative studies described in Table 1. In a 2020 multicenter randomized controlled trial comparing EUS-GBD using LAMS and PCT in 80 subjects with acute cholecystitis, patients who underwent EUS-GBD had a significantly lower adverse event rate at one year, with rates of 25.6% compared to 77.5% in the PCT group (*p* = 0.001). Additionally, only 2.6% of EUS-GBD patients required re-intervention after 30 days due to stent occlusion. In contrast, 30% of patients in the PCT intervention group required tube re-insertion (*p* = 0.001). Notably, in 60% of patients who received LAMS, gallstones drained freely through the gastrointestinal lumen. Some patients underwent subsequent endoscopy for cholecystoscopy through the LAMS and ensuing stone removal [46]. 

### 4.5. EUS-GBD: Complications

The potential complications of abnormal stent migration and biliary leak are decreased with the use of LAMS with double-pigtail stent placed through the metal stent as compared to SEMS, plastic stents and nasobiliary drains alone [39,50]. Placing a stent in the gastric antrum leads to higher risk of stent occlusion from food debris as compared to duodenal deployment [7]. Less common side effects can include duodenal or gallbladder perforation, biliary pain, excess bleeding, self-limiting pneumoperitoneum, bile leak, and peritonitis from stent migration [17,39,40]. One large systematic review that included 17 EUS-GBD studies found that adverse events occurred in 11.7% of LAMS cases, some of which included bleeding, infection, bile leak, pain, stent migration, perforation, pneumoperitoneum, and recurrent cholecystitis [39,51,52]. Despite these potential complications associated with LAMS, Table 2 highlights the favorable profile of EUS-GBD when compared to ETP-GBD in technical success, clinical success, adverse events, rates of recurrent intervention, and rates of recurrent cholecystitis.

## 5. Choosing between ETP-GBD and EUS-GBD

### 5.1. Main Considerations

In cases where a patient is considered high-risk for surgical cholecystectomy, lacks evidence of gallbladder perforation, and can safely undergo sedation, the current AGA recommendation is to first explore EUS-GBD or ETP-GBD before considering PCT. If there is evidence of ascites, or a need for ERCP to manage choledocholithiasis, or if the gallbladder is more than 1 cm away from the duodenum or stomach, then ETP-GBD is the preferred choice. If it is expected that the patient will be a future surgical candidate, experts recommend discussing with a surgeon to determine if they would be comfortable performing cholecystectomy if EUS-GBD is performed, as the fistula from LAMS can make surgery more challenging [9]. Surgeons may favor the transgastric LAMS for EUS-GBD if there is future possibility of cholecystectomy, as closing a gastric defect is often less complex than closing one in the duodenum [6]. Table 3 further describes a list of benefits and limitations when comparing EUS-GBD and ETP-GBD.

If ETP-GBD does not need to be completed for the aforementioned reasons, recent data has emerged demonstrating the advantage of EUS-GBD over ETP-GBD despite similar technical and clinical success rates in comparison studies. This preference for EUS-GBD stems from shorter hospital stays, shorter time for clinical improvement, and lower rates of required reintervention [9,48]. In addition, EUS-GBD is recommended if there is gastric outlet obstruction or duodenal obstruction, blockage of the cystic duct, or a substantial quantity of gallstones are present [9]. 

Conventionally, endoscopists have avoided EUS-GBD in individuals with gallbladder perforation. However, one recently published case report involved an elderly lady who presented with acute perforated cholecystitis and was deemed unfit for surgery due to medical comorbidities. A 10 mm × 10 mm transgastric LAMS was placed successfully with resolution of cholecystitis, and the patient remained symptom free two months post-procedure. This case highlights how many EUS-GBD listed contraindications may be theoretical and relative rather than absolute due to scarcity of data [56].

In the instance there is an adverse event after EUS-GBD or ETP-GBD, the treatment involves correcting the underlying cause of the complication or closely monitoring the patient until it resolves. For example, one study found self-limiting pneumoperitoneum that resolved with conservative management in 3.4% of EUS-GBD patients [17]. Likewise, post-ERCP pancreatitis is typically managed expectantly. LAMS occlusion, bleeding, abnormal stent migration, or bile leak is often managed through repeat endoscopy [6,17]. Creating a hole in the gallbladder in anticipation of EUS-GBD could lead to bile leak if the procedure is unsuccessful, which can be treated with ETP-GBD and stent placement, PCT, or surgery [57]. Hence, if one method of non-surgical gallbladder drainage fails, the others are typically pursued. 

### 5.2. Stone Removal through LAMS via EUS-GBD and Additional Review of Literature Comparing EUS-GBD, ETP-GBD, and PCT

LAMS placement with EUS-GBD allows for endoscopic removal of smaller gallstones from the gallbladder that can easily pass through the saddle portion of the stent. At high volume institutions, patients routinely undergo cholecystoscopy one month following EUS-GBD for stone removal, LAMS removal, and placement of a double pigtail plastic stent [58]. Recent studies have emerged demonstrating that large stones can be removed through both endoscopic laser lithotripsy and lithotomy (ELLL) and electrohydraulic lithotripsy (EHL) [59,60]. In one study by Wang et al., a gallstone as large as 23.8 × 28.8 mm was successfully extracted through the ELLL method after it was broken into pieces smaller than 1 cm. They initiated patients on regular supplementation of ursodeoxycholic acid to prevent re-formation of gallstones, with no recurrence of lithiasis 6 months post-ELLL [59]. Several authors have demonstrated large gallstone disintegration through EHL and subsequent extraction through LAMS, with smaller stones removed via washing, and larger particles removed with a retrieval basket [60,61]. Most data on this use of ELLL and EHL is through case reports, and larger trials are needed. 

A recent systematic review and meta-analysis comparing EUS-GBD, ETP-GBD, and PCT to our comprised of 1267 patients contained ten studies that met inclusion criteria. In this review, the authors determined that EUS-GBD had the highest rates of clinical success, ETP-GBD had the largest risk of postprocedural adverse events, EUS-GBD had the smallest degree of recurrent cholecystitis, PCT had the largest amount of recurrent interventions and unplanned admissions, while all-cause mortality was the least with ETP-GBD. After interpretation of their results, the authors concluded that EUS-GBD is the optimal treatment for patients who will not be future surgical candidates [62].

### 5.3. Cost Considerations 

Although EUS-GBD is typically recommended over ETP-GBD for endoscopic management of acute cholecystitis, there is an associated upfront cost. The only fully covered electrocautery-enhanced LAMS currently FDA approved and available in the US is the AXIOS stent from Boston Scientific (Marlborough, MA, USA), which is approximately $5000, with a total EUS procedure cost estimated at $5600 [40,63]. This is compared to the price of a plastic pigtail stent, estimated at $50, with a total ETP-GBD procedure cost of $1900 [40]. There are several other LAMS available worldwide: Spaxus (Taewoong Medical, Los Angeles, CA, USA), Nagi (Taewoong Medical), Hanarostent Plumber (M.I. Tech, Houghton, MI, USA), and Aixstent (Leufen Medical, Berlin, Germany) [64]. It is unclear if these other LAMS could enter the US market in the future at a lower cost. In addition, there are potential cost savings when taking into account the decreased hospital length of stay when patients receive EUS-GBD as compared to ETP-GBD and PCT [51].

## 6. Conclusions

Endoscopic gallbladder drainage for acute cholecystitis is becoming more common and is an increasingly preferred approach for nonsurgical patients over PCT. EUS-GBD is favored over ETP-GBD if feasible. Although LAMS was previously only approved for pancreatic pseudocyst drainage, the FDA recently permitted marketing of the AXIOS stent for gallbladder drainage in August 2023, which may enhance its adoption. The most recent FDA-approved clinical trial evaluating EUS-GBD was published in 2023 and demonstrated clinical improvement in all patients who successfully received LAMS placement. A transduodenal approach is preferred when possible due to lower risk of complications such as stent occlusion [45]. Careful selection of patients is important. Technical and clinical success is higher when the procedures are performed by experienced endoscopists ideally at high-volume centers. In the future, additional trials comparing EUS-GBD or ETP-GBD to PCT may help reinforce the clinical success of patients treated endoscopically for acute cholecystitis. 

## Figures and Tables

**Table 1:** Chosen studies comparing Endoscopic Ultrasound-Guided Gallbladder Drainage (EUS-GBD) to Percutaneous Transhepatic Cholangiography (PCT).

Study	N	Technique	Technical Success (%)	Clinical Success (%)	Adverse Events (%)	Total Number of Recurrent Interventions (%)	Comments
Tyberg 2018 [47]	42	EUS-GBD	95.2%	95.2%	21.4%	11.9%	Adverse events and reinterventions were followed over a 71 month period.
	113	PCT	99.1%	85.8%	21.2%	39.8%	
Irani 2017 [48]	45	EUS-GBD	97.8%	95.6%	17.8%	24.4%	Median follow up period 215 vs. 265 days, Study reported adverse events and re-intervention over this entire window.
	45	PCT	100.0%	91.1%	31.1%	248.9%	
Teoh 2020 [46]	39	EUS-GBD	97.4%	92.3%	12.8%	2.6%	30-day adverse event rate. Reinterventions after 30 days rate.
	40	PCT	100.0%	92.5%	47.5%	30.0%	
Jang 2012 [49]	30	EUS-GBD	96.7%	96.7%	6.7%		Adverse event periprocedural, recurrent intervention not reported.
	29	PCT	96.6%	93.1%	3.4%		

**Table 2:** Studies comparing the efficacy of Endoscopic Ultrasound-Guided Gallbladder Drainage (EUS-GBD) and Endoscopic Transpapillary Gallbladder Drainage (ETP-GBD).

Study	N	Technique	Technical Success (%)	Clinical Success (%)	Adverse Events (%)	Recurrent Intervention (%)	Recurrent Cholecystitis (%)	Comments
Oh 2019 [39]	76	EUS-GBD	99.3%	99.3%	7.1%	3.9%	3.6%	Recurrent intervention performed for recurrent cholecystits or cholangitis, some events of stent migration did not have a subsequent intervention.
	96	ETP-GBD	86.6%	86.0%	19.3%	17.4%	10.4%	
Higa 2019 [40]	40	EUS-GBD	97.5%	95.0%	17.9%	17.9%	2.5%	
	38	ETP-GBD	87.2%	76.3%	9.4%	21.9%	15.8%	
Teeratorn 2019 [53]	17	EUS-GBD	100.0%	100.0%	5.8%		7.7%	Reintervention rates not reported.
	83	ETP-GBD	78.3%	73.4%	6.0%		4.8%	
Siddiqui 2019 [51]	102	EUS-GBD	94.1%	90.2%	11.8%	0.0%	1.0%	
	124	ETP-GBD	87.9%	79.8%	7.3%	11.4%	3.2%	
Matsubara 2016 [54]	21	EUS-GBD	100.0%	90.5%	19.0%		0.0%	Reintervention rates not reported.
	257	ETP-GBD	77.4%	72.4%	16.3%		8.9%	
Inoue 2023 [55]	90	EUS-GBD	96.7%	88.9%	12.2%		3.8%	
	90	ETP-GBD	78.9%	74.4%	21.1%		3.0%	Reintervention rates not reported.

**Table 3:** Advantages and Limitations of Endoscopic Ultrasound-Guided Gallbladder Drainage (EUS-GBD) and Endoscopic Transpapillary Gallbladder Drainage (ETP-GBD).

	Benefits	Limitations
EUS-GBD	Lower risk of recurrent cholecystitis Lower rate of reintervention Can drain gallstones through stent Ability to provide tract for cholecystoscopy and lithotripsy of gallstones Stent can remain in place permanently In appropriate candidate, preferred by AGA in 2023 guidelines	Technically challenging to perform Lower rates of technical success Cost of LAMS Risk of obstruction Potential difficulties with surgical cholecystectomy due to fistula Limited reported use with gallbladder perforation Caution regarding use with ascites
ETP-GBD	Preferentially used in individuals needing ERCP for choledocholithiasis or another indication High rates of technical success Able to perform if gallbladder is positioned far from duodenal or gastric lumen Preferred procedure with large volume ascites	Higher risk of recurrent cholecystitis ERCP related risks including pancreatitis
PCT	Frequently performed and easily accessible High rates of technical success Close follow-up required for recall procedure or device removal	High rates of post-procedure adverse events High rates of required reintervention Higher rate of readmission External drain can provide inconvenience Not recommended with large volume ascites
